# Muscle Derived Mesenchymal Stem Cells Inhibit the Activity of the Free and the Neutrophil Extracellular Trap (NET)-Bond Myeloperoxidase

**DOI:** 10.3390/cells10123486

**Published:** 2021-12-10

**Authors:** Thierry Franck, Justine Ceusters, Hélène Graide, Ange Mouithys-Mickalad, Didier Serteyn

**Affiliations:** 1 Centre of Oxygen Research and Development (CORD), University of Liege, 4000 Liege, Belgium; J.Ceusters@uliege.be (J.C.); helene.graide@revatis.com (H.G.); amouithys@uliege.be (A.M.-M.); didier.serteyn@uliege.be (D.S.); 2 Research Unit FARAH, Department of Clinical Sciences, Faculty of Veterinary Medicine, University of Liege, 4000 Liege, Belgium

**Keywords:** muscle derived mesenchymal stem cells, neutrophils, ROS, myeloperoxidase activity, NET

## Abstract

Mesenchymal stem cells (MSCs) are known to migrate to tissue injury sites to participate in immune modulation, tissue remodelling and wound healing, reducing tissue damage. Upon neutrophil activation, there is a release of myeloperoxidase (MPO), an oxidant enzyme. But little is known about the direct role of MSCs on MPO activity. The aim of this study was to investigate the effect of equine mesenchymal stem cells derived from muscle microinvasive biopsy (mdMSC) on the oxidant response of neutrophils and particularly on the activity of the myeloperoxidase released by stimulated equine neutrophils. After specific treatment (trypsin and washings in phosphate buffer saline), the mdMSCs were exposed to isolated neutrophils. The effect of the suspended mdMSCs was studied on the ROS production and the release of total and active MPO by stimulated neutrophils and specifically on the activity of MPO in a neutrophil-free model. Additionally, we developed a model combining adherent mdMSCs with neutrophils to study total and active MPO from the neutrophil extracellular trap (NET). Our results show that mdMSCs inhibited the ROS production, the activity of MPO released by stimulated neutrophils and the activity of MPO bound to the NET. Moreover, the co-incubation of mdMSCs directly with MPO results in a strong inhibition of the peroxidase activity of MPO, probably by affecting the active site of the enzyme. We confirm the strong potential of mdMSCs to lower the oxidant response of neutrophils. The novelty of our study is an evident inhibition of the activity of MPO by MSCs. The results indicated a new potential therapeutic approach of mdMSCs in the inhibition of MPO, which is considered as a pro-oxidant actor in numerous chronic and acute inflammatory pathologies.

## 1. Introduction

In addition to their well-known regenerative properties, mesenchymal stem cells (MSCs) exhibit anti-inflammatory properties which have led to their increasing therapeutic use [1,2]. MSCs are known to migrate to tissue damage sites, where they participate in tissue remodelling, wound healing and modulation of the immune response [3]. The paracrine activity appeared as a predominant mechanism by which MSCs participate in tissue repair instead of engraftment and differentiation into functional cells [4,5] and has been shown to decrease angiogenesis and apoptosis and to modulate extracellular matrix dynamics and inflammation [3]. MSCs influence the immune system through anti-inflammatory properties and modulate the activity and function of macrophages and neutrophils [6]. MSCs have been shown to reduce the tissue damage induced by neutrophils [7]. A recent study showed that modulation of the redox environment and oxidative stress by MSCs can mediate their anti-inflammatory and cytoprotective properties and offer potential antioxidant mechanisms for MSCs therapies [8]. In a model of acute endotoxin (LPS)-induced lung inflammation in mice, MSCs reduced inflammation and inhibited the formation of neutrophil extracellular traps (NET) [9]. The influence of MSCs in adherent or in suspension condition including their supernatants was already studied on the neutrophil oxidative metabolism, including the ROS production, phagocytosis, apoptosis, and NET formation in animal and human cell models [10,11,12], including horse [13]. 

However, neutrophils, during degranulation, release an important pro-oxidant enzyme, myeloperoxidase (MPO), which is involved in the oxidation of many biomolecules by its dual activity of peroxidase and chlorination; this last activity being responsible for the formation of the strong oxidant molecule HOCl [14,15]. The oxidative potential of MPO is normally used inside and outside the neutrophil to fight invasion of microorganisms, but in acute and chronic inflammation the uncontrolled release of MPO into the extracellular medium can exacerbate inflammation and tissue damage while playing a crucial role in biomolecule transformation [15]. Few studies have been performed on the potential role of MSCs on MPO released by neutrophil degranulation and on their enzymatic activity. In disease models with MSC injection, MPO is often taken as a marker of neutrophil infiltration, localisation or expression during in vitro or ex vitro studies [8,16]. In some in vitro models, the activity of MPO is measured as marker of neutrophil degranulation [6,7,17] but few studies concern the potential inhibitory effect of MSCs directly on the activity of MPO. Moreover, MSCs derived from adipose tissue or bone marrow are generally used, but MSCs from skeletal muscle origin are not. As far back in 2010, skeletal muscle was considered as a reservoir for cells with properties that are similar to those of mesenchymal stem cells [18]. Recently, a study showed that human MSCs derived from skeletal muscle have superior biological properties compared to trabecular bone-derived cells [19]. In 2017, Ceusters et al. [20] developed a novel method to obtain mesenchymal stem cells derived from muscle microbiopsies (mdMSCs) of mammalian origin. The technique provides a minimally invasive methodology for producing large amounts of mdMSCs showing, among other qualities, interesting differentiation properties (Patent WO/2015/091210) [21]. In the context of a future therapeutic use, the cells were washed two times and suspended in sterile phosphate buffer saline (PBS).

The aim of this work was to study the effect of equine mdMSCs harvested in PBS on the oxidative response of neutrophils not only on ROS production, but also to focus their effect on the release of total and active MPO during in vitro degranulation of stimulated neutrophils. The effect of isolated mdMSCs was studied directly on the activity of MPO using a technique that enables the collection of evidence of the binding of an inhibitor with the enzyme. Finally, washed mdMSCs were loaded in culture medium and left to adhere before adding neutrophils to study active and total MPO bound to NET. Regarding our results, an immunological technique was proposed to assay specifically total and active MPO bound to NET formed after neutrophil stimulation.

## 2. Material and Methods

### 2.1. Chemicals and Reagents

Analytical grade phosphate salts, sodium and potassium chloride, sodium hydroxide, sodium acetate, H_2_O_2_ (30%) and Tween 20, Percoll (GE Healthcare), T-flasks, conical bottom centrifuge tubes were purchased from Merck (VWR International, Leuven, Belgium). The 8-amino-5-chloro-7-phenylpyrido[3,4-d]pyridazine-1,4(2H,3H)dione (L-012) was purchased from Fujifilm Wako Chemicals Europe GmbH (Neuss, Germany). The bovine serum albumin fraction V (BSA) was obtained from Roche Diagnostics (Mannheim, Germany). The phorbol 12-myristate 13-acetate (PMA), cytochalasin B, N-formyl-methionyl-leucyl-phenylalanine (fMLP), sodium nitrite, were purchased from Sigma-Aldrich (Bornem, Belgium). The 96-well microtiter plates (Combiplate 8 EB) and 96-well white plates, the fluorogenic substrate, Amplex red (10-acetyl-3,7-dihydroxyphenoxazine) (Invitrogen), trypsin TrypLE Express (Gibco, Thermo Fisher Scientific, Waltham, MA, USA) and Hank’s balanced salt solution (HBSS) 1× (Gibco, Thermo Fisher Scientific, Waltham, MA, USA) and Fetal Bovine Serum (FBS) were purchased from Fischer Scientific (Merelbeke, Belgium). The Dulbecco’s Modified Eagle Medium Ham’s F12 (DMEM F12) culture medium with Hepes and glutamine, penicillin-streptomycin, amphotericin B and the Dulbecco’s phosphate buffer saline (DPBS) were purchased from Lonza (Verviers, Belgium). The equine MPO ELISA kit was purchased from BiopTis (Vielsalm, Belgium). The purified equine neutrophil MPO was obtained as previously described [22], with the following characteristics: 70.4 U/mg as specific activity and 3.38 mg/mL as protein concentration. The rabbit and guinea pig antibodies against equine MPO were purchased from Bioptis (Vielsalm, Belgium). The rabbit polyclonal antibodies to citrullinated Histone H3 (citrulline R2 + R8 + R17) were purchased from Abcam (Cambridge, UK).

### 2.2. Equine Muscle-Derived Mesenchymal Stem Cells (mdMSC) Culture and Sample Cell Preparation

The equine mdMSCs were obtained from skeletal muscle micro-biopsies and the methods to isolate and characterize them were published in Ceusters et al. [20]. A summary of the methodology for obtaining mdMSCs and their characterization is included in the Supplementary Material. Micro-biopsies were sampled from 4 horses from the Horse European Centre of Mont-le-Soie (Vielsalm, Belgium) and the isolation, culture, and cold storage of mdMSCs were processed by Revatis (Aye, Belgium). The cells were cultured in DMEM F-12 culture medium supplemented with 20% heat-inactivated fetal bovine serum (HI-FBS), 1% penicillin (1000 U/mL)-streptomycin (10,000 µg/mL) and 0.5% of amphotericin B at 37 °C and 5% CO_2_. All the experiments were conducted with cells in culture between passages five and seven, according to our previous studies [20]. Cells were seeded in 175 cm^2^ culture flasks. For each experiment, cells from 4 T-flasks at 75–80% confluency were generally used. The culture medium was removed, then the cells were rinsed with 5 mL DPBS and incubated for 5 min at 37 °C with 4 mL of synthetic trypsin (TrypLE Express; Gibco). Thereafter, 6 mL of DPBS were added and the detached cells from the 4 T-flasks were transferred in a 50 mL tube for centrifugation (330× *g*, 10 min, 37 °C). The cell pellet was resuspended in 5 mL of DPBS and centrifuged again (330× *g*, 10 min, 37 °C). The final pellet was resuspended in DPBS, the cells were counted under light microscopy and their viability was determined by the Trypan blue exclusion technique [23]. The cells obtained in DPBS were diluted in DPBS, HBSS or medium according to the experimental protocols.

### 2.3. Effects of mdMSCs on the ROS Production by Neutrophils

Equine neutrophils used in all experiments were isolated from whole blood drawn from five healthy horses from the Equine Clinic of Liege University (Liège, Belgium). These horses are dedicated for student formation and observation. The neutrophils were isolated according to the method described by Pycock et al. [24] and suspended in DPBS before activation. Cell preparation contained ≥ 95% neutrophils. Smears of neutrophils isolated from two horses were performed on the microscope slide, stained by Diff-Quik (Medion Diagnostics) and observed by light microscopy (Zeiss, Axioskop). Based on 5 different microscopic fields per horse (100 cells/field), the purity of the neutrophil population was estimated to be 97.1 ± 0.9% neutrophils. The other cells are eosinophils.

The neutrophils (0.5 × 10^6^ neutrophils/well) were incubated for 10 min in the wells of a white microtiter plate with various number of isolated mdMSCs in DPBS buffer. Dilutions of the cell suspension and reagents were performed to work with a final volume of 200 µL. The superoxide anion production was measured by chemiluminescence (CL) according to Franck et al. [25], but L-012 was used as a chemiluminescent probe. L-012 is a luminol analogue generally used to measure reactive oxygen and nitrogen species (RNOS) in inflammatory situations, especially superoxide anions derived from NADPH oxidase activity [26]; it is also used to detect HOCl formation resulting from MPO activity [27].

Before the measurement, 10 μL of L-012 (1.2 mg/mL in distilled water) and 10 μL of phorbol 12-myristate-13-acetate (PMA) (16 μM in 1% DMSO in ultra-pure H_2_O) were added. Just after PMA addition, the CL response was monitored for 30 min and expressed as the integral value of total CL emission. Two controls were performed, one with the PMA-activated neutrophils without mdMSCs, and another with non-stimulated neutrophils and without mdMSCS where PMA was replaced by its vehicle solution (1% DMSO in H_2_O).

### 2.4. Effects of mdMSCs on the Active and Total MPO Release by Neutrophil Degranulation

Neutrophils suspended in DPBS (1 × 10^6^ cells) in 2 mL tube were mixed with increasing number of mdMSCs in DPBS and incubated during 30 min at 37 °C. Dilutions of the cell suspensions and reagents were performed in order to work with a final volume of 1 mL. After incubation, 2 µL of a cytochalasin B solution (2 mg/mL in DMSO) was added to each tube and a second incubation was performed for 30 min at 37 °C, followed by the addition of 10 µL of a 10^−4^ M fMLP solution (in 10% DMSO ultra-pure water). After 1h incubation at 37 °C, the tubes were centrifuged (350× *g*, 10 min, 22 °C) and the supernatants were collected and frozen at −20 °C before MPO assay. Two controls were performed with only PMNs, activated and not activated, each receiving the equivalent volumes of the vehicle solution of cytochalasin B and fMLP (control PMN A and control PMN NA).

An ELISA assay kit (Equine MPO ELISA, BiopTis, Vielsalm, Belgium) was used to measure the total equine MPO concentration in the collected supernatants, which were diluted 200× with 20 mM DPBS before the assays.

The active MPO released in the supernatants was measured by the SIEFED method that we have developed for the specific detection of active neutrophil MPO in complex biological samples [28]. The SIEFED method follows a three-step procedure: firstly, the extraction of the enzyme from the sample by its capture on specific immobilized antibodies (3 µg/mL rabbit anti-equine MPO antibodies); secondly, washings to eliminate non-specific bound compounds or interfering substances; and, thirdly, the detection of the MPO activity after addition of H_2_O_2_ as substrate, using Amplex red as fluorogenic electron donor and nitrite as an enhancer of the reaction.

In more detail, the peroxidase activity of MPO was monitored by adding 100 μL of a 40 μM Amplex red solution freshly prepared in 50 mM phosphate buffer, pH 7.4, supplemented with 10 μM H_2_O_2_ and 10 mM sodium nitrite. Fluorescence development was monitored during 30 min (37 °C) with a Fluoroskan Ascent (Thermo Fisher Scientific, Waltham, MA, USA) set at 544 nm and 590 nm for the excitation and emission wavelengths, respectively. Total fluorescence was directly proportional to the amount of active MPO present in the sample. For this assay, the samples were not diluted.

### 2.5. Measurement of the Direct Effects of mdMSCs on the Activity of Isolated MPO

The SIEFED assay was used as a pharmacological tool for the screening of MPO inhibitors [25]. One million mdMSCs were suspended in 1 mL HBSS and serial dilutions were performed to obtain cell suspensions ranging from 100,000 to 195 cells in 500 µL HBSS. Each cell dilution was added to 500 µL of a 10 mU/mL equine MPO solution in HBSS to reach a final 5 mU of MPO in contact with the cells. The control assay was performed in the same experimental conditions but with 500 µL HBSS without mdMSCs. All the samples were loaded in duplicate into the wells of a microplate precoated with rabbit anti-equine MPO antibodies (3 µg/mL). After a new incubation (2 h, 37 °C), the wells were carefully emptied, and the plate was drained on a paper towel to remove any remaining liquid. The wells containing the captured MPO were washed four times with PBS solution containing 0.1% Tween 20. Finally, the peroxidase activity of MPO was monitored by adding 100 μL of a 40 μM Amplex red solution freshly prepared in 50 mM phosphate buffer, pH 7.4, supplemented with 10 μM H_2_O_2_ and 10 mM sodium nitrite. The fluorescence development was monitored during 30 min (37 °C) with a Fluoroskan Ascent (Thermo Fisher Scientific, Waltham, MA, USA) set at 544 nm and 590 nm for the excitation and emission wavelengths, respectively. Total fluorescence was directly proportional to the amount of active MPO present in the sample.

### 2.6. Effects of mdMSCs on the NET Formation after Equine Neutrophils Stimulation

#### 2.6.1. Sample Preparation

After culture, detachment by trypsination and washing in DPBS as described above, the mdMSCs (0.500, 0.250, 0.125, 0.062 and 0.031 × 10^6^ cells) were loaded in six well cell culture plates (Cellstar, Greiner bio one, Vilvoorde, Belgium) in 2 mL of DMEM F-12 medium supplemented with 20% FBS and incubated 24 h at 37 °C and 5% CO_2_. The control wells were filled with the culture medium without mdMSCs. After incubation, the medium was removed, and the wells were gentle rinsed with 1 mL DPBS. After the aspiration of DPBS, a neutrophil suspension in DMEM F12 medium with 20% FBS was added to the wells (1 million PMNs in 2 mL medium by well). After a new incubation for 30 min at 37 °C and 5% CO_2_, 4 µL of a 2 mg/mL cytochalasin B solution in DMSO was added to each well except in the control wells, which contained only PMNs and to which 4 µL DMSO was added. The plate was incubated again for 30 min at 37 °C and 5% CO_2_. Then, 20 µL of a 10^−4^ M fMLP solution (in 10% DMS0 ultra-pure water) was added to each well except the non-stimulated neutrophil control well, which received 10 µL of the fMLP vehicle solution. The plate was incubated 24 h at 37 °C and 5% CO_2_ before a gentle mixing of the cell suspension of each well by pipetting up and down the medium 3 times. The medium was then transferred to 2 mL tubes, centrifuged (350× *g*, 10 min, 22 °C) and the supernatant was collected and frozen at −20 °C before the NET assay.

#### 2.6.2. Assay of Total and Active MPO Bound to the NET (NET-MPO)

The NET released by the stimulated neutrophils was captured by anti-histone H3 (citrulline R2 + R8 + R17; anti-H3Cit) antibodies based on the method described by Thalin et al. [29]. After NET capture, the presence of active MPO bound to the NET was detected by the SIEFED technique.

Transparent 96-wells microplates were coated overnight, at 4 °C, with 100 μL/well rabbit anti-H3Cit (0.5 μg/mL) diluted with 20 mM PBS buffer. After removal of the coating solution, the plates were incubated (150 min, 22 °C) with 200 µL blocking buffer (PBS buffer with 5 g/L of BSA) then washed four times with the washing solution (PBS buffer with 0.1% Tween 20). The plates were dried for 3 h at 22 °C, then conserved in a dry atmosphere in a hermetic bag at 4 °C until use. The supernatants dedicated to the NET assay were loaded (100 µL) in duplicate into the wells of the anti-H3Cit coated microplate and incubated 2 h at 37 °C. After incubation, the supernatants were removed, and the wells were washed four times with a PBS solution containing 0.1% Tween 20 before active or total MPO was measured.

The activity of MPO bound to the NET was measured by the revelation of the peroxidase activity of MPO as described above by adding sodium nitrite and Amplex red solution, then by monitoring fluorescence development over 30 min.

The total MPO from the NET was detected by using a second anti-MPO antibody (guinea pig antibody coupled to alkaline phosphatase) and the revelation technique from the ELISA assay kit (Equine MPO ELISA, BiopTis, Vielsalm, Belgium).

#### 2.6.3. Assay of H3Cit Bound to the NET (NET-H3Cit)

The NET released by the stimulated neutrophils was captured by anti-MPO antibodies. After NET capture, the presence of H3Cit was detected using anti-histone H3 (citrulline R2 + R8 + R17).

Transparent 96-well microplates were coated overnight, at 4 °C, with 100 μL/well guinea pig anti MPO (2 μg/mL) diluted with 20 mM PBS buffer according to the technique described above. The supernatants dedicated to the NET assay were loaded (100 µL) in duplicate into the wells of the anti-MPO coated microplate and incubated 2 h at 37 °C. After incubation, the supernatants were removed, and the wells were washed four times with a PBS solution containing 0.1% Tween 20. Then, 100 µL/well of rabbit anti-H3Cit solution (1 µg/mL) prepared in PBS buffer with 0.1% Tween 20 and 1% BSA was added and incubated 2 h at 37 °C. After incubation, the antibody solution and the wells were washed four times with a PBS solution. After washing, a third antibody produced in goat against rabbit IgG and labelled with alkaline phosphatase (Abcam, Cambridge, UK) was added to recognize the H3Cit antibody. After incubation (2 h, 37 °C), the unbond third antibody was eliminated by 4 washings with PBS solution containing 0.1% Tween 20, and then a ready-to-use paranitrophenyl phosphate solution (D-tek, Mons, Belgium) was added to the well and incubated for 30 min at 37 °C in the dark. The reaction was stopped with 2.5 M NaOH and the absorbance (405 nm) was read with the Multiskan Ascent plate reader (Thermo Fisher Scientific, Waltham, MA, USA). The absorbance value was directly proportional to the quantity of the antiMPO–MPO–antiH3Cit sandwich complex.

### 2.7. Statistical Analysis

Experiments were made with cell batches from 4 horses for mdMSCs isolation and culture, and from 5 horses for blood sampling and neutrophil isolation. In some experiments, mdMSCs from the same horse were tested twice with neutrophil batches from two different horses. Each point in the experiment was performed at least in duplicate. Five independent experiments were taken into consideration for the figures and statistical analysis.

For figures, the data are expressed as mean ± standard deviation (SD) and given in relative values (%) in reference to control groups taken as 100%.

For the statistical analysis, the raw data were used. The mean of each technical replicate was calculated for each horse and the means and the SDs obtained from the five independent experiments were considered for the statistical analysis (*n* = 5).

Since some populations or data series did not follow a Gaussian distribution, a non-parametric test was used: a two tail Mann–Whitney test was performed (Graph Pad, InStat, San, Diego, CA, USA). A *p*-value < 0.05 was considered significant.

## 3. Results

### 3.1. Effect of mdMSCs on ROS Production by Stimulated PMNs

**Figure 1:**
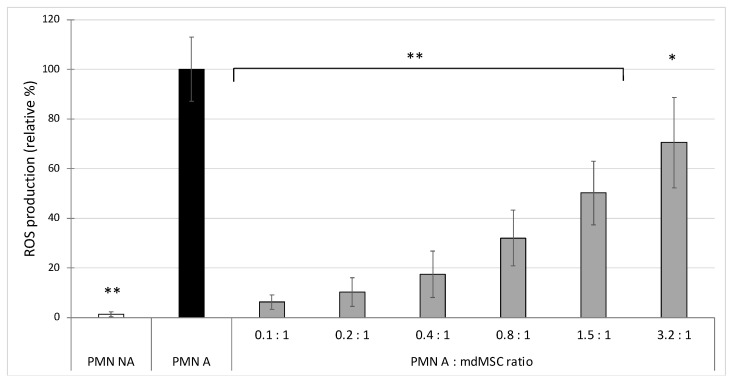
Effect of non-activated (PMN NA) and PMA-activated neutrophils (PMN A) alone or mixed with mdMSCs at different ratios (PMN A: mdMSC ratio) in DPBS on ROS production. Results from five independent experiments (*n* = 5) with 2 technical replicates for each point, including 4 horses for the mdMSCs and 5 horses for the neutrophils. Means ± SD are shown in relative percentages versus stimulated neutrophils without mdMSCs (PMN A), defined as 100% response. Statistical analysis was considered for 5 independent experiments ** *p* < 0.01, * *p* < 0.05 versus PMN A.

The data were obtained from 5 independent experiments performed with different neutrophils and mdMSCs preparations. The stimulation of PMNs induced an intense ROS production in comparison to the non-activated PMNs (Figure 1). The addition of mdMSCs significantly inhibited, in a dose dependent manner, this ROS production. From PMN:mdMSC ratios of 0.1:1 to 3.2:1, a significant inhibition was observed ranging from 29 ± 18% to 94 ± 3%, respectively, in comparison to stimulated PMNs alone (Figure 1).

### 3.2. Effect of mdMSCs on MPO Degranulation

**Figure 2:**
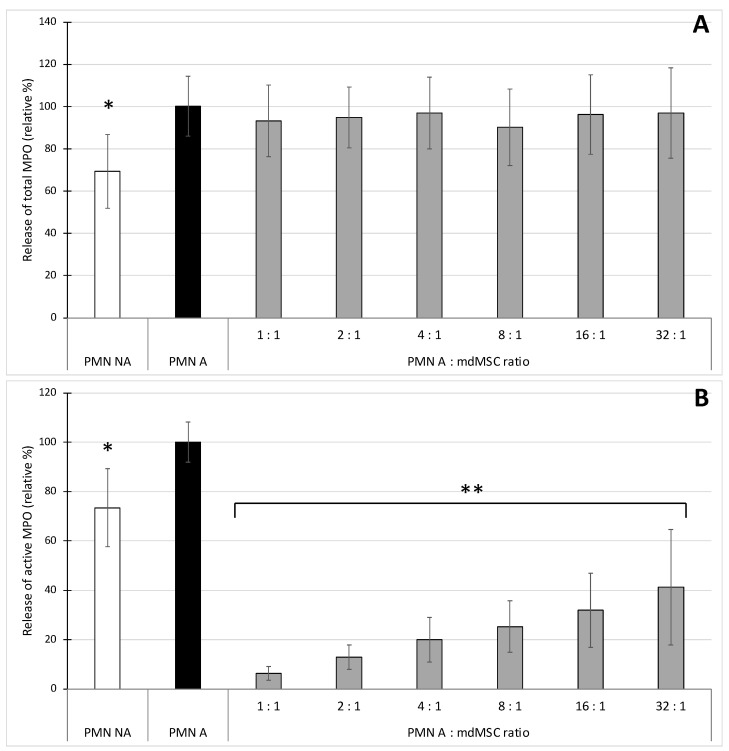
Effect of non-activated (PMN NA) and CB/fMLP-activated neutrophils (PMN A) alone or mixed with mdMSCs at different ratios (PMN A:mdMSC ratio) in DPBS on the degranulation of total (**A**) and active MPO (**B**). Results from five independent experiments (*n* = 5) with 2 technical replicates for each point, including 4 horses for mdMSCs, 5 horses for neutrophils. Means ± SD are shown in relative percentages versus stimulated neutrophils alone defined as 100% response (PMN A). Statistical analysis was considered for 5 independent experiments ** *p* < 0.01, * *p* < 0.05 vs. PMN A.

For total MPO (measured by ELISA), the stimulation of PMNs with CB/fMLP induced a moderate but significant (* *p* < 0.05) release of MPO compared to non-stimulated conditions, and the presence of mdMSCs did not significantly disturb the release of total MPO by stimulated PMNs (Figure 2A).

Concerning the release of active MPO (measured by SIEFED), the stimulation of PMNs also induced a moderate but significant (* *p* < 0.05) increase of active MPO in comparison to non-stimulated conditions. The presence of mdMSCs inhibited, dose-dependently, the release of active MPO by the stimulated neutrophils. From PMN A:mdMSC ratios of 1:1 to 32:1, the inhibition appeared significant (** *p* < 0.01) and ranged from 59 ± 23% to 94 ± 3% (Figure 2B).

### 3.3. Effect of MSCs on MPO Activity

**Figure 3:**
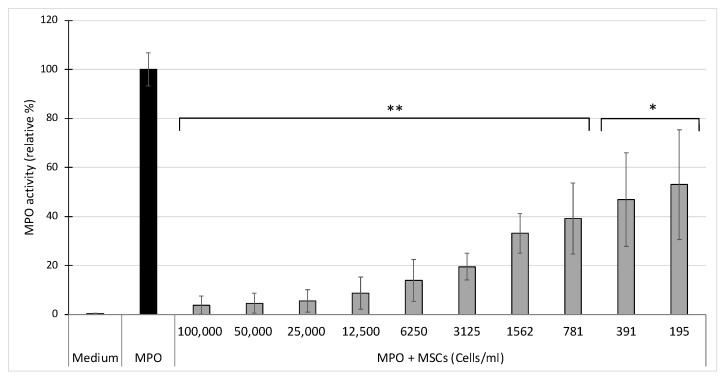
Effect of MSCs on the activity of equine MPO measured by SIEFED. Results from five independent experiments (*n* = 5) with 2 technical replicates for each point, including 4 horses for mdMSCs. Means ± SD are shown in relative percentages versus MPO control performed without mdMSCs and defined as 100% response. Statistical analysis was considered for 5 independent experiments ** *p* < 0.01, * *p* < 0.05 vs. MPO.

Using the SIEFED technique, we showed that mdMSCS induce a strong dose dependent inhibition of purified MPO activity in a neutrophil-free system (Figure 3). According to the statistical analysis, the inhibition appeared very significant (** *p* < 0.01) from the 781 cells/mL level and significant * *p* < 0.05 for 195 and 391 cells/mL. The inhibition reached about 90% from the 12,500 cells/mL and about 50% from 195 cells/mL levels, in comparison to the medium containing only MPO and set as 100% response.

### 3.4. Effect of mdMSCs on the NET-Bound MPO Release by Stimulated PMNs

**Figure 4:**
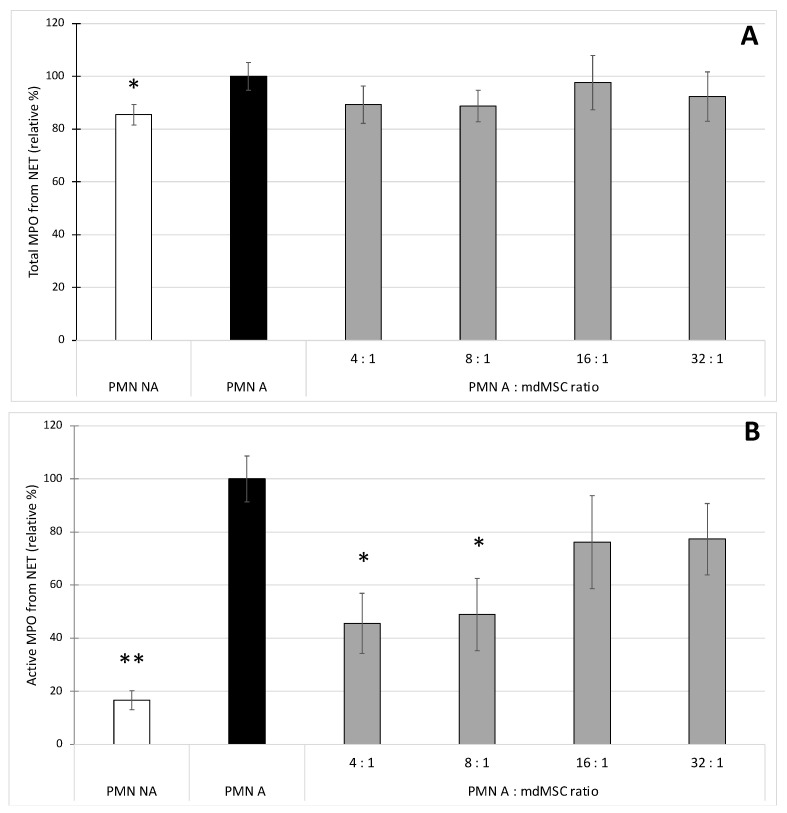
Effect of non-activated (PMN NA) and CB/fMLP-activated neutrophils (PMN A) alone or in coculture with mdMSCs at different ratios (PMN A:mdMSC ratio) on total (**A**) and active (**B**) MPO attached to the NET. Results from five independent experiments (*n* = 5) with 2 technical replicates for each point, including 4 horses for mdMSCs, 5 horses for neutrophils. Means ± SD are shown in relative percentages versus stimulated neutrophils without mdMSCs (PMN A) defined as 100% response. Statistical analysis was considered for 5 independent experiments ** *p* < 0.01, * *p* < 0.05 vs. PMN A.

The activation of PMNs with CB/fMLP induced a slight but significant increase (* *p* < 0.05) of total NET-MPO (about 15%) in comparison to non-stimulated PMNs. When PMNs were stimulated in presence of mdMSCs, the PMN A:mdMSC ratios of 4:1 and 8:1 induced a slight (about 10%), but not significant decrease of the total NET-MPO in comparison to PMNs stimulated alone (Figure 4A).

When studying the active MPO bound to the NET, the stimulation of PMNs induced a highly significant increase (** *p* < 0.01) of NET-MPO activity (about 80%) in comparison to non-stimulated conditions. Mixtures of stimulated PMNs with mdMSC at 4:1 and 8:1 ratios induced a significant decrease (* *p* < 0.05) of the NET-MPO activity in comparison to PMNs stimulated alone. A decrease of 51 ± 14% was observed from the 8:1 ratio (Figure 4B).

### 3.5. Effect of mdMSCs on the NET-Bound H3Cit Release by Stimulated PMNs

**Figure 5:**
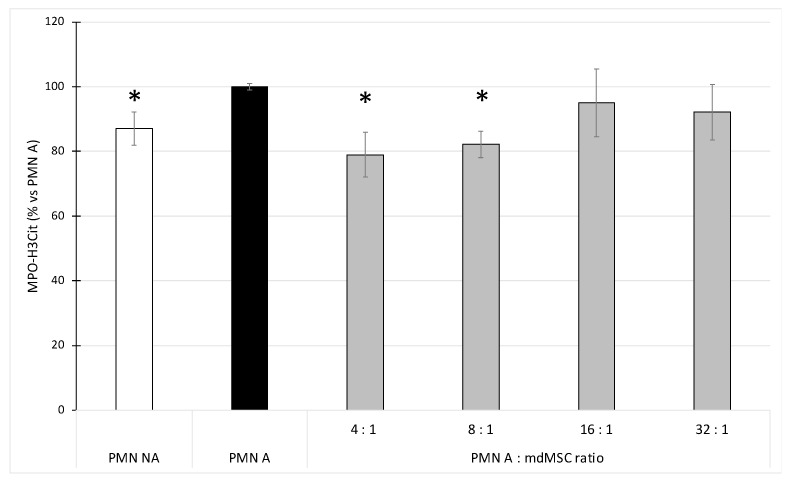
Effect of non-activated (PMN NA) and CB/fMLP-activated neutrophils (PMN A) alone or in coculture with mdMSCs at different ratios (PMN A:mdMSC ratio) on H3Cit associated to MPO-DNA complexes of the NET. Results from 5 independent experiments (*n* = 5) with 2 technical replicates for each point, including 4 horses for mdMSCs and 5 horses for neutrophils. Means ± SD are shown in relative percentages versus stimulated neutrophils without mdMSCs (PMN A) defined as 100% response. Statistical analysis was considered for 5 independent experiments * *p* < 0.05 vs. PMN A.

As for total NET-MPO (Figure 4A), the activation of PMNs with CB/fMLP induced a slight but significant increase (* *p* < 0.05) of H3Cit associated to MPO-DNA complexes (about 15%), in comparison to non-stimulated PMNs (Figure 5). When PMNs were stimulated in the presence of mdMSCs, the PMN A:mdMSC ratios of 4:1 and 8:1 induced a slight (about 20%) but significant decrease (* *p* < 0.05) of the NET-bound H3Cit in comparison to PMNs stimulated alone (Figure 5).

## 4. Discussion

In most studies dealing with the inhibitory potential of mesenchymal stem cells on the neutrophil oxidative metabolism, MSCs were in adherent cultured conditions or in suspension in their culture medium [10,11,12,13]. In the context of a future therapeutic use of equine mesenchymal stem cells derived from micro-invasive muscle biopsy [20], we tried to minimise interference from the cell culture medium before in vitro or in vivo use. For this purpose, a protocol of cell multiplication and preparation was established, which consists of culturing the cells in adherent conditions in T-flasks, harvesting them with trypsin in DPBS and, after centrifugation, washing them 2 times with DPBS before their direct use or their freezing in a cryopreservation medium. This process was used for the autologous production of stem cells dedicated to several clinical applications in veterinary medicine and for research in human medicine (Revatis SA, Aye, Belgium). Therefore, in our models, we used isolated mdMSCs washed two times with DPBS before experiments.

In the first experiment, we observed that the co-presence of mdMSCs with isolated neutrophils in DPBS inhibits, in a dose dependent manner, ROS production by PMA stimulated neutrophils, by using L-012 as a chemiluminescent probe (Figure 1). Compared to other studies showing a significant inhibitory effect on ROS production with high neutrophil:MSC ratios (10:1, 100:1 and even 1000:1) [7,11,13], we obtained a significant inhibition with a less favourable neutrophil/MSC ratio (3.2:1). Indeed, these studies used MSCs preincubated 24 h into the medium before neutrophil addition, or mixed MSCs and neutrophils in a culture medium for 4 h before their stimulation. However, under our experimental conditions, the cells were suspended in PBS as if they were going to be used for clinical application and were mixed with neutrophils 10 min before their stimulation, which could explain our low ratio compared to those of other studies, which favoured a much longer contact time between neutrophils and MSCs suspension. Nevertheless, our results suggest that cells conditioned in PBS before transplantation had or have acquired very early an inhibitory capacity on the oxidant response of neutrophils.

According to Stavely and Nurgaly [8], MSCs can directly dampen the respiratory burst in neutrophils. A part of the antioxidant response of stem cells could be cell contact dependent [8], but paracrine activity appeared to play a fundamental role. The antioxidant compounds and antioxidant enzymes that regulate the redox status are found in the exosomes and extracellular vesicles released by MSCs including miRNAs, catalase, SOD1, SOD2, glutathione peroxidase. [8,30,31,32]. Cell to cell contact or paracrine activity would interfere with the NADPH assembly at the membrane level, as suggested by Sharma et al. [33], and also with the degranulation process responsible for MPO release. Neutrophil stimulation with PMA involves a high production of superoxide anions which can alter MPO activity, making this activation model not ideal for studying MPO activity [34,35].

In a second set of experiments (Figure 2), we stimulated neutrophils in the presence of mdMSCs with the combination CB/fMLP, mimicking a more physiological process for neutrophil stimulation and degranulation [35]. Such stimulating conditions did not induce an important increase of total MPO release that could be attributed to the absence of calcium into DPBS [36]. However, other experiments performed in Hank’s Balanced Salt Solution (HBSS) containing calcium (results not shown) or in complete medium (see Figure 4 and Figure 5) did not improve the release of total MPO during stimulation, suggesting a calcium-independent response in our conditions. The results showed that the presence of mdMSCs did not reduce the total MPO released by stimulated PMNs, and that the activity of the released enzyme was inhibited related to the number of cells, suggesting a potential role of mdMSCs on MPO activity with a neutrophil:MSC ratio about 10 times higher (32:1) than that observed for ROS production. In contradiction with our results, an inhibition effect of mesenchymal stromal cells or adipose-derived stem cells was previously observed on the total MPO released by stimulated neutrophils [6,7]. However, these authors used adherent or suspended cells in the presence of their culture medium.

By measuring the peroxidase activity of MPO as a marker of phagocyte degranulation, Jiang et al. [7] and Guillén et al. [17] have shown that MSCs decreased the degranulation of stimulated neutrophils and monocytes respectively. With the SIEFED technique specifically used for MPO activity measurement in parallel with a classical ELISA detecting the total amount of released MPO, we demonstrated that mdMSCs did not affect the total amount of MPO released by the phagocytes. Our results suggest that mdMSCs would not significantly affect the degranulation process, but would be active to limit the oxidative stress linked to the release of active MPO by activated neutrophils. Guillén et al. [17] noted the potential of MSCs in inhibiting MPO to control oxidative stress. To confirm the potential inhibitory role of mdMSCs directly on MPO activity, we used the SIEFED technique as a pharmacological tool that we have designed to find MPO inhibitors interacting directly with the enzyme [37]. The mdMSCs were incubated for 2 h at 37 °C with purified equine MPO in the well of a microtiter plate coated with MPO antibodies. After incubation, mdMSCs were eliminated by washings and the activity of MPO captured by the antibodies was revealed. Despite the washing of the cells, a significant inhibition of MPO activity was observed compared to the control test performed with MPO alone (Figure 3). We verified that this inhibition was not due to a lack of MPO binding to antibodies due to the presence of cells by measuring the total MPO content using ELISA and found the same total amount of MPO in the MPO control and the cell samples with MPO (results not shown). Thus, our results suggest that the mdMSCs had released a compound lowering or blocking the MPO activity. Because the sample was eliminated by washings before the enzymatic revelation, it means that the compound released by the cells acted directly on the enzyme, maybe on its catalytic site, and not on the oxidant species formed by MPO. The nature of this inhibition needs to be further determined. Since this inhibition is linked to a direct interaction with MPO, proteins or lipids lost from the membrane of mdMSCs could be incriminated, but microvesicles could hinder the access of the substrates to the enzymatic site of MPO. Indeed, MSCs have the capacity to secrete multiple paracrine factors, either soluble or associated to microvesicles. The microvesicles released by mesenchymal stem cells are enriched in miRNAs endowed with remarkable antioxidant activity: they increase ferric ion-reducing antioxidant ability by increasing the activity of antioxidant enzymes and they decrease reactive oxygen species (ROS) generation, DNA/lipid/protein oxidation, and stress-associated molecular patterns in cultured cells [30,38]. In our study, the use of mdMSCs after several washings in DPBS may have influenced the paracrine capacity of the cells and favoured the release of free soluble molecules, lipids, peptides or proteins, which could either enter directly in the active site of the enzyme if they are small enough or could obstruct the access channel to the active site if their size is too large.

MPO appears as a major element of the NET, and is necessary for its formation by contributing to protease activation and their release in the nuclei, allowing histone degradation and chromatin decondensation [39]. Circulating MPO-DNA complexes are increasingly used as a marker of NET levels in various diseases [40,41]. According to Jiang et al. [7,42], we developed a methodology to study the effect of mdMSCs on NET formation which consists of allowing mdMSCs to adhere to the culture plate before adding neutrophils and stimulating them. Contrary to authors who used PMA as a neutrophil activator, we adopted the combination of CB/fMLP already used for studying MPO degranulation by neutrophils mixed with mdMSCs in suspension. The early stages of NET formation involve the enzyme peptidylarginine deiminase 4 (PAD4) responsible for histone citrullination. Several studies considered citrullinated histone as a marker of NET, leading to the development of several immunological assays using anti-histone 3 antibodies [29,43]. In our study, the rabbit polyclonal anti-histone H3 (citrulline R2 + R8 + R17) used to detect NET in the human plasma sample [29] was used in two experimental manners. First, it was coated onto a microplate to capture the NET present in our sample. After washing to eliminate the unbound compounds of the sample, the presence of the NET was revealed indirectly by measurement of the presence of the total concentration or specifically the active fraction of MPO attached to the NET. Second, the anti-histone H3 (citrulline R2 + R8 + R17) was used to detect H3Cit from MPO-DNA complexes captured by the anti-MPO polyclonal antibody. Considering the total MPO content bound to NET (Figure 4A) and the H3Cit content bound to NET (Figure 5) as two markers of NET presence, we observed very similar results between the two assays, which validates the specificity of MPO-H3Cit complexes for NET detection in our model. Preliminary results obtained by qPCR (Progenus Company, Gembloux, Belgium) confirmed the presence of nuclear DNA and traces of mitochondrial DNA on the MPO-H3Cit complexes released after their capture by anti-H3Cit antibodies, attesting that our immunological technique allows the capture of MPO-H3Cit complexes associated to a DNA network.

Our results showed that unstimulated PMNs incubated in the culture medium of mdMSCs caused a basal NET release which slightly enhances upon PMN stimulation with CB/fMLP (Figure 4A and Figure 5). The copresence of stimulated neutrophils and mdMSCs with ratios of 4:1 and 8:1 allowed a slight but significant decrease of the NET release. The presence of mdMSCs will probably downregulate NET formation by the early inhibition of intracellular ROS production and MPO activity, both necessary to trigger NET release from neutrophils [44]. It was shown that pharmacological MPO inhibitors can decrease NET formation, although the process is not fully blocked, probably because the inhibitors do not reach the granules [44], which seems to be in accordance with our results. However, regarding the active MPO, the stimulation of neutrophils allowed the measurement of a higher activity of MPO attached to the NET, and the presence of mdMSCs substantially decreased the activity of the NET-MPO. To our knowledge, these observations have never been reported and they highlight a potential inhibitory effect of mdMSCs on the active MPO attached to the NET even after its formation.

In summary, we show that equine mdMSCs submitted to a process involving several washings with DPBS keep an important capacity to inhibit the oxidant response of neutrophils. Our results highlight the important role of mdMSCs on the direct inhibition of a highly oxidant enzyme, myeloperoxidase, involved in several processes particularly linked to the oxidative response of neutrophils, including ROS production, degranulation and NET formation. Our data also offer evidence of the important presence of active MPO attached to the NET released by stimulated neutrophils with the ability of mdMSCs to inhibit the catalytic activity of NET-MPO. Finally, our work underlines a new potential therapeutic approach of mdMSCs in the inhibition of MPO, which is now recognized as an important actor in numerous chronic and acute inflammatory pathologies.

## Supplementary Materials

The following are available online at https://www.mdpi.com/article/10.3390/cells10123486/s1, Annex 1: Muscle derived stem cells.
