# Supplementary material for: Muscle Derived Mesenchymal Stem Cells Inhibit the Activity of the Free and the Neutrophil Extracellular Trap (NET)-Bond Myeloperoxidase
Source: Cells. 2021 Dec 10;10(12):3486. doi: 10.3390/cells10123486 (PMC8700239; doi:10.3390/cells10123486)
Supplement: Supplementary file 1 [file cells-10-03486-s001.zip › cells-1443851-supplementary.pdf]

Supplementary materials

Muscle derived stem cells (mdMSCs)

Muscle-derived stem cells (mdMSCs) were isolated according to a patented method (Mammalian muscle-derived stem cells, WO/2015/091210) and as described by Ceusters et al (2017).

Briefly, microbiopsy specimens (approximately 15 to 20 mg of tissue) were obtained from triceps brachii muscles (long chief) with a semi-automated 14-gauge microbiopsy pistol and placed in culture medium (DF20) composed of 500 mL DMEM - Ham's F12 (Gibco, Thermo Fisher scientific, Belgium) supplemented with 100mL of Foetal Bovin Serum (FBS) heat inactivated from South America (Gibco, Thermo Fisher scientific, Belgium), 5mL of penicillin/streptomycin (Gibco, Thermo Fisher scientific, Belgium) and 2,5mL of amphotericin B (Gibco, Thermo Fisher scientific, Belgium). Microbiopsies were cut in a Petri dish into 16 pieces, placed in the 16 central wells of a 24-well plate (Greiner, Bio-One, Germany) and 150µl of DF20 were added to each well whereas the outer wells were filled with PBS (1ml/well). Then, culture dishes were incubated at 37°C under controlled atmosphere (5% CO_2 and 21% O_2) till a halo of cells was visible around the piece of muscle.

The mdMSCs were isolated from cells obtained from explants. The cells were harvested using TrypLE (Gibco, Thermo Fisher scientific, Belgium), centrifuged (200 x g, 10 min, 37°C) and the pellet was suspended in 1ml of HBSS (Gibco, Thermo Fisher scientific, Belgium). The cellular suspension was then placed on a 3 layers discontinuous Percoll density gradient (15%, 25% and 35%) and centrifuged at 1250 x g (25°C, 20min). The 15-25% fraction was collected, washed once with HBSS and centrifuged at 200 x g, 10 min at 37°C. The supernatant was discarded and the pellet was suspended in 1ml of DF20. The cells were first cultured in T-25 cm² flask (Nunc, VWR, Belgium) until 80% confluence. They were finally multiply in T-175 cm² flask (Nunc, VWR, Belgium) till obtaining a sufficient amount of cells.

The mdMSCs were characterised regarding their ability to differentiate into the 3 lineages, *ie* adipocytes, chondrocytes and osteocytes, while placing them in the adequate differentiation medium. Their immunophenotyping was also assessed by flow cytometry regarding their expression of CD90 and CD44 (>90%) and non-expression of CD45 and HLA—DR (<2%). Results are presented in detail in Ceusters et al. (2017)
